# SARS-CoV-2 induced hepatic injuries and liver complications

**DOI:** 10.3389/fcimb.2022.726263

**Published:** 2022-09-16

**Authors:** Umar Saeed, Zahra Zahid Piracha, Sara Rizwan Uppal, Yasir Waheed, Rizwan Uppal

**Affiliations:** ^1^ Department of Research and Development, Islamabad Diagnostic Center (IDC), Islamabad, Pakistan; ^2^ International Center of Medical Sciences Research(ICMSR), Islamabad, Pakistan; ^3^ Department of ORIC, Shaheed Zulfiqar Ali Bhutto Medical University, Islamabad, Pakistan

**Keywords:** SARS-CoV-2, COVID-19, liver injury, hepatocellular carcinoma, hepatic dysfunction, mechanism of liver damage, immunopathogenesis

## Abstract

**Background:**

Coronavirus disease 2019 (COVID-19) is caused by severe acute respiratory syndrome coronavirus-2 (SARS-CoV-2), which is resilient, highly pathogenic, and rapidly transmissible. COVID-19 patients have been reported to have underlying chronic liver abnormalities linked to hepatic dysfunction.

**Discussion:**

Viral RNAs are detectable in fecal samples by RT-PCR even after negative respiratory samples, which suggests that SARS-CoV-2 can affect the gastrointestinal tract and the liver. The case fatality rates are higher among the elderly and those with underlying comorbidities such as hypertension, diabetes, liver abnormality, and heart disease. There is insufficient research on signaling pathways. Identification of molecular mechanisms involved in SARS-CoV-2-induced damages to hepatocytes is challenging. Herein, we demonstrated the multifactorial effects of SARS-CoV-2 on liver injury such as psychological stress, immunopathogenesis, systemic inflammation, ischemia and hypoxia, drug toxicity, antibody-dependent enhancement (ADE) of infection, and several others which can significantly damage the liver.

**Conclusion:**

During the COVID-19 pandemic, it is necessary for clinicians across the globe to pay attention to SARS-CoV-2-mediated liver injury to manage the rising burden of hepatocellular carcinoma. To face the challenges during the resumption of clinical services for patients with pre-existing liver abnormalities and HCC, the impact of SARS-CoV-2 on hepatocytes should be investigated both *in vitro* and *in vivo*.

## Introduction

Coronaviruses are enveloped, single-stranded RNA viruses belonging to *Coronaviridae*. The human coronaviruses such as severe acute respiratory syndrome coronavirus (SARS-CoV), SARS-CoV-2, and MERS-CoV are highly pathogenic; however, others such as HCoV-HKU1, HCoV-229E, HCoV-OC43, and HCoV-NL63 are less virulent. Electron microscopy revealed a crown (halo) around the virus, which explains the coronavirus nomenclature. The infections are common among mammals and birds. The zoonotic infections by *Coronaviridae* affect predominantly the cells in the upper respiratory tract (Li, 2015). Genome sequence analysis revealed 80% homology of SARS-CoV-2 with the bat coronavirus (Jothimani et al., 2020).

The global coronavirus disease 2019 (COVID-19) pandemic caused SARS-CoV-2 infection among 497 million people and caused 6.17 million deaths (WHO). The SARS-CoV-2 infection rate varies across the globe due to the emergence of multiple variants. The United States of America, despite having world-class healthcare facilities, remained the top among the worst-affected countries with 80.3 million infections and 0.98 million deaths (WHO). A multicenter analysis revealed that among 838 SARS-CoV-2-positive patients, 51.2% were reported with liver injuries and hepatic abnormalities. It has been suggested that possibly 28.2% of deaths were reported due to cholestasis patterns and 25% of deaths were due to hepatocellular injuries followed by 22.3% of deaths due to mixed patterns (Chu et al., 2020).

SARS-CoV-2 contains four major structural proteins, namely, nucleocapsid, matrix core, envelop, and glycoprotein spike surface proteins (Saeed et al., 2021). SARS-CoV-2 binds to the angiotensin‐converting enzyme 2 (ACE2) receptor, which is expressed on AT2 human epithelial cells. The virus penetrates the host cell through clathrin- and caveolae-independent endocytic pathways and *via* the host cell-directed network of G-protein-coupled receptors, and it may activate c-Jun N-terminal Kinase (JNK) and Janus Tyrosine Kinase (JAK)-Signal Transducer and Activator of Transcription (STAT) pathways for enhanced viral replication (Konrad et al., 2020).

COVID-19 infection symptoms include nasal congestion, runny nose, sore throat, and diarrhea. Severe patients often suffer from dyspnea and/or hypoxemia 1 week after onset, and these patients rapidly progress to acute respiratory distress syndrome, septic shock, intractable metabolic acidosis, and coagulation disorders. SARS-CoV-2-associated liver injury occurs possibly due to multiple factors such as direct cytopathic effect of SARS-CoV-2 *via* the ACE2 receptor, ischemia hypoxia and circulatory changes, hepatotoxic effect of drugs, inflammatory responses, viral-induced cytotoxic T cells, and pre-existing liver disease. Patients with pre-existing chronic hepatic diseases or hepatocellular carcinoma (HCC) are vulnerable to COVID-19-induced liver dysfunction. Reactivation of liver-targeting viruses such as hepatitis B virus may also trigger liver failure (Aldhaleei et al., 2020).

## Insight into SARS-CoV-2 causing COVID-19 disease progression

The SARS-CoV-2 gets access to the host cell *via* the spike (S) protein of 180 kDa. The amino (N) terminal region of S contributes to binding to ACE2, while the carboxyl (C) terminal region contributes to the fusion of viral and cellular membranes (Li, 2015). The polybasic furin cleavage site in the spike protein (which is absent in SARS-CoV) and the expression of ACE2 and other cellular proteases (such as TMPRSS2) also contribute to cellular tropism (Andersen et al., 2020). After furin cleavage, the spike protein RXXR motif binds to the NRP1 receptor which promotes viral infection through unknown mechanisms, which needs further investigation (Cantuti-Castelvetri et al., 2020). ACE functions as a vasopressor and performs pro-inflammatory roles (Cantuti-Castelvetri et al., 2020; Magro et al., 2020). The ACE converts angiotensin I to angiotensin II, and through its carboxy peptidase activity, it generates heptapeptide angiotensin-1 to angiotensin-7. In addition to the expression of ACE2, there might be several other factors that contribute to SARS-CoV-2 infection progression, which needs further analysis.

During the presymptomatic/asymptomatic phase, SARS-CoV-2 infects ACE2-expressing nasal epithelial cells in the upper respiratory tract. During the early phase, the virus infects ACE2-expressing type II alveolar epithelial cells, and patients exhibit pneumonitis. Meanwhile, during the late phase (days 7 to 10), severe disease involves disruption of the epithelial–endothelial barrier, complement deposition, and hyperinflammation. Severe systemic effects of SARS-CoV-2 might be due to a series of events such as loss of pulmonary epithelial–endothelial cell integrity (immune-driven inflammation might trigger pulmonary endothelial injury *via* complement activation and/or cytokine release causing rupture to the pulmonary epithelial–endothelial barrier which aggravates endothelial damage and promotes the transmission of SARS-CoV-2) and septal capillary injury, followed by neutrophil infiltration, complement deposition, intravascular viral antigen deposition, and localized intravascular coagulation (Gavriilaki et al., 2020; Magro et al., 2020; Matheson and Lehner, 2020).

According to the Human Cell Atlas, the expression of ACE2 was higher in intestinal enterocytes as compared to the lungs, which suggests the non-enzymatic functions of ACE2 (like chaperoning amino acid transporters). About 30% of COVID-19 patients had detectable viral RNA in their stool samples, speculating the SARS-CoV-2 effects on the gastrointestinal (GI) tract which might contribute to GI abnormalities among the patients (Magro et al., 2020). Since innate and adaptive immune responses might trigger systemic inflammatory responses, the expression of the spike protein of SARS-CoV was linked to liver inflammation.

## Immunopathogenesis and etiology of liver injury

During stress, immune tolerance is disturbed. Uncontrolled immune responses may trigger immunopathogenesis that consequently causes lung tissue damage and functional impairment (Li et al., 2020). Upon SARS-CoV infection, CD4^+^ T cells provide helper functions to B cells and initiate antibody production. Higher levels of cytotoxic CD8^+^ T lymphocytes were linked to pulmonary injuries, which further aggravate hyperactive immune responses, inducing cytokine storm and, consequently, systemic inflammation (Li et al., 2020).

Upon SARS-CoV-2 infection, the peripheral blood levels of interleukins 2, 6, 7, and 10; interferon-inducible protein 10; granulocyte colony-stimulating factor; macrophage inflammatory protein 1 alpha; ferritin; tumor necrosis factor-alpha; monocyte chemotactic protein 1; CD8^+^ T cells; and Th17 were significantly enhanced (Cai et al., 2020; Diao et al., 2020; Huang et al., 2020; Liu et al., 2020; Wan et al., 2020). Oxidative stress, hyperactivation of Kupffer cells, and sympathetic nervous and adrenocortical system activation due to hypoxia–reoxygenation were linked to metabolic acidosis, calcium overloading, and loss of mitochondrial membrane permeability causing sepsis-related liver injuries in COVID-19 patients (Li and Fan, 2020).

## Ischemia and hypoxia–reperfusion dysfunction

Oxidative stress marked the elevation of reactive oxygen species (ROS) and their peroxidation (as a second messenger) may stimulate several redox-sensitive transcription factors that trigger multiple proinflammatory factors, consequently causing hepatic injury (Zhang et al., 2018). Hypoxia- and shock-induced inflammatory cell infiltration may lead to hepatic cell death (Yang et al., 2019). Ischemia and hypoxia can cause glycogen consumption, lipid accumulation, excretion of toxic metabolites, and depletion of adenosine triphosphate, inhibiting signal transduction and cell survival (Yang et al., 2019). These lines of evidence demonstrate that pneumonia-associated hypoxia is one of the most critical factors causing secondary liver injury among patients with severe and critical COVID-19 disease.

## Enhancement of SARS-CoV-2 infection due to antibodies and associated immune complexes

The distinct mechanisms of viral infection *via* antibody‐dependent enhancement include antibody-mediated virus uptake into Fc gamma receptor IIa (FcγRIIa)-expressing phagocytic cells and enhanced antibody Fc-mediated effector functions.

The Fc receptor (FcR) and/or complement receptor (CR) of a virus-specific antibody can interact to enhance viral access to granulocytes, monocytes, and macrophages. This phenomenon aggravates the proliferation of viral progeny and may cause adverse infection. It has been reported that SARS-CoV-2 spike-specific antibodies caused the antibody-dependent enhancement (ADE) of infection in ACE2 non-expressing immune cells and caused severe damage to hepatocytes (Wang et al., 2014).

The enhanced antibody Fc-mediated effector functions may also cause aggressive immunopathology and inflammation. Post-SARS-CoV-2 systemic infection, the formation of antigen–antibody immune complexes might lead to the deposition of immune complexes promoting vascular damage and systemic inflammation (Wang et al., 2014). Identifying the distinct mechanisms of ADE infection could be valuable for the safety analysis of SARS-CoV-2-specific future vaccines.

## Hepatic histopathological alterations due to SARS-CoV-2

In addition to hepatitis viruses, liver injuries were also reported due to other viruses such as yellow fever, dengue, MERS, influenza, and SARS (Chang et al., 2006; Cao et al., 2009; Saad et al., 2014; Costa et al., 2019). Several distinct factors were reported with viruses such as SARS-CoV-2 mode of action, repercussion of systemic hemodynamic abnormalities, inflammatory alterations, and coagulation disorders that may induce liver injuries indicated by histopathological changes (Sonzogni et al., 2020; Tian et al., 2020; Xu et al., 2020). Postmortem studies of SARS-CoV-2-infected deceased patients revealed abnormal intrahepatic blood vessel networks and histopathological alterations (Sonzogni et al., 2020). Furthermore, vascular abnormalities were reported possibly due to enhanced hepatic arterial flow leading to secondary cardiac distress and thrombotic phenomena of portal and sinusoidal vessels (Sonzogni et al., 2020).

## Non-alcoholic fatty liver disease and non-alcoholic steatohepatitis due to COVID-19

Non-alcoholic fatty liver disease (NAFLD) is a liver metabolic syndrome, and its aggressive form is non-alcoholic steatohepatitis (NASH). NAFLD syndrome patients are more prone to SARS-CoV-2 infection. Multiple studies supported the evidence that the expression of ACE2 was higher in adipose tissue than in pulmonary tissue. It has been reported that obese patients had higher viral shedding time and were associated with respiratory distress and correlated with mechanical ventilation (Ji et al., 2020). Body mass index (BMI) and obesity were related to the overall survival of COVID-19 patients (Cai et al., 2020; Kassir, 2020). Among SARS-CoV-2 patients, serum monocyte chemoattractant protein-1 chemokine levels were significantly elevated and were responsible for exacerbating steatohepatitis (Boeckmans et al., 2020). Further studies are needed for an accurate investigation regarding the effects of SARS-CoV-2 on NAFLD and NASH.

## Liver function tests for abnormalities and consequent liver injuries related to SARS-CoV-2

In SARS-CoV-2-infected individuals, liver function can be assessed by monitoring the dysregulation of multiple factors including alanine aminotransferase (ALT), alkaline phosphatase (ALP), aspartate aminotransferase (AST), gamma-glutamyl transferase (GGT), total bilirubin, prolonged prothrombin time, and several others (Meyer et al., 2020). Among SARS-CoV-2 patients, 76.3% showed abnormal liver test results (Cai et al., 2020). The severity of COVID-19 disease progression among SARS-CoV-2-infected individuals was significantly correlated with higher serum levels of ALT, AST, and total bilirubin and lower serum levels of albumin (Parohan et al., 2020), and 62% of COVID-19 ICU patients had elevated levels of AST (Huang et al., 2020). Also, the serum levels of GGT were increased up to 72% among SARS-CoV-2-positive patients (Wan et al., 2020). Furthermore, another study reported significantly higher levels of ALT and AST among severe as compared to non-severe patients (Guan et al., 2020).

## Hepatotoxic drugs and COVID-19

In the absence of established treatment regimens against SAR-CoV-2, several antibiotics, antivirals, antipyretics, analgesics, and traditional Chinese medicines were empirically used in an attempt to save lives. However, the possible side effects and serious adverse effects of those drugs remained unnoticed for a period of time, raising questions about patient safety and management.

Angiotensin II receptor blockers and ACE inhibitor drugs have protective effects against SARS-CoV-2 (Diao et al., 2020). Lopinavir/ritonavir usage among SARS-CoV-2 patients showed 55.4% of drug-induced liver injuries (Diao et al., 2020). The general medications prescribed for SARS-CoV-2 such as oseltamivir, lopinavir/ritonavir, and chloroquine are metabolized in the liver (Rismanbaf and Zarei, 2020). Overdose of antipyretic drugs (such as paracetamol) caused liver injury. Also, hydroxychloroquine has been reported to cause acute liver failure (Makin et al., 1994). The usage of macrolide antibiotics such as azithromycin was also linked to liver injury (Martinez et al., 2015). Methylprednisolone (to alleviate SARS-CoV-2-associated cytokine storm) and tocilizumab (targeting interleukin-6 receptor) may also aggravate liver cirrhosis (Liu et al., 2020), and remdesivir was significantly correlated with the upregulation of liver enzymes (Zampino et al., 2020).

## Conclusion

**Figure 1:**
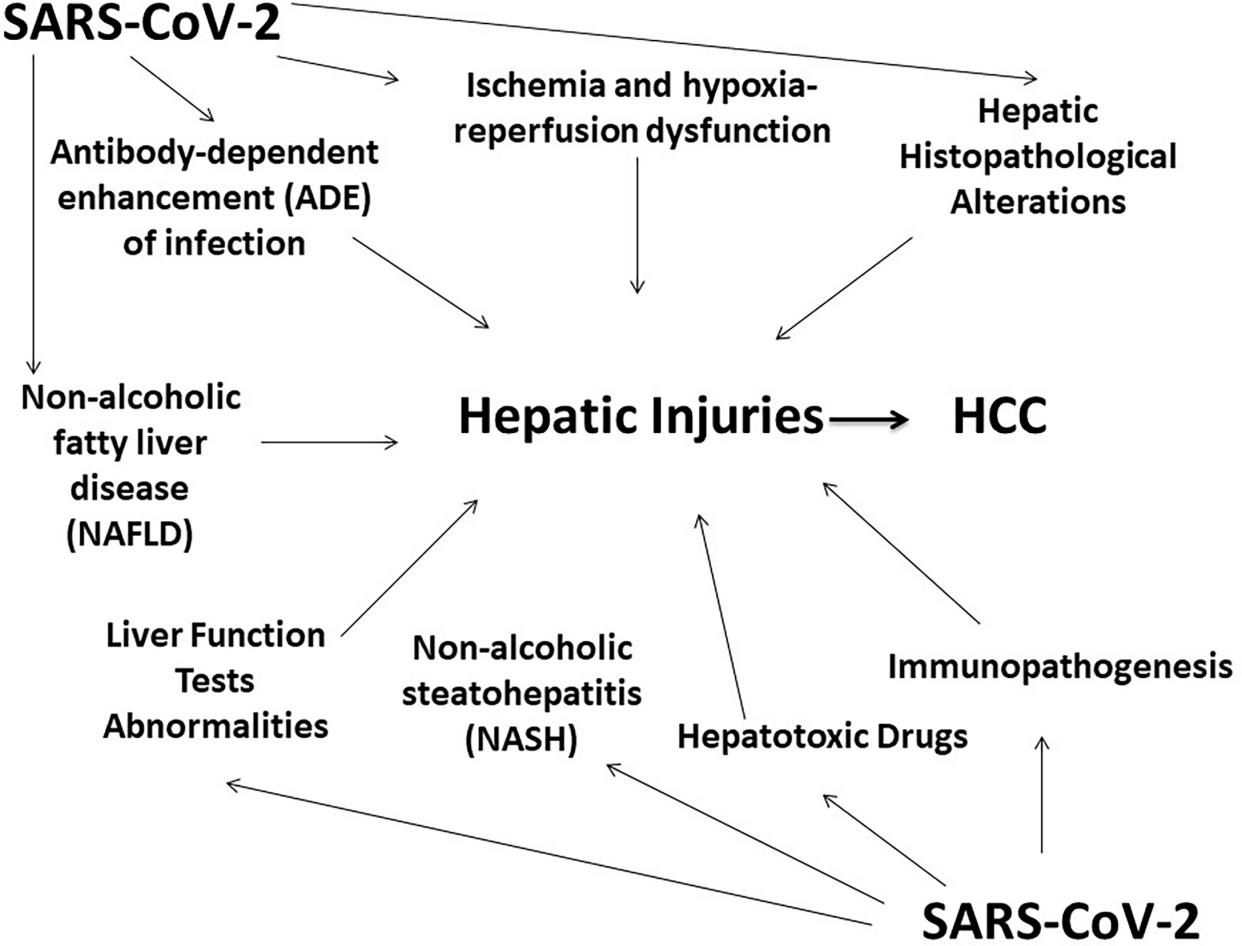
SARS-CoV-2 and Liver injuries.

Viral–host intermediate interaction and signaling are critical for contemplating disease progression (Saeed et al., 2014; Saeed et al., 2017; Piracha et al., 2018; Saeed et al., 2019; Piracha et al., 2020; Saeed et al., 2021). The underlying mechanisms of liver injuries due to COVID-19 might include several factors such as psychological stress, drug toxicity, progression of pre-existing liver abnormalities, SARS-CoV-2 infection of hepatocytes, immune system complications, or a cytokine storm (**Figure 1**). A mechanistic understanding of the relationship between COVID-19 and liver complications is needed for improving treatment options for SARS-COV-2-infected patients. Several mechanistic studies are also needed to elucidate SAR-CoV-2-associated cytopathic effects, hepatocyte damage, and consequent liver injuries. The clinical management of patients with COVID-19-associated liver damage is a challenging task and needs more attention. Intensive surveillance approaches should be tailored for immune-compromised patients with HCC and for liver transplant patients, with further research warranted in this area.

## Author contributions

US conceived the study, wrote the manuscript, and analyzed the data. RU is the principal investigator (PI) of the study and US is the co-PI of the study. ZP, SU, YW and RU contributed to manuscript revision and data analysis. All authors contributed to the article and approved the submitted version.

## Conflict of interest

The authors declare that the research was conducted in the absence of any commercial or financial relationships that could be construed as a potential conflict of interest.

## Publisher’s note

All claims expressed in this article are solely those of the authors and do not necessarily represent those of their affiliated organizations, or those of the publisher, the editors and the reviewers. Any product that may be evaluated in this article, or claim that may be made by its manufacturer, is not guaranteed or endorsed by the publisher.

## Abbreviations

SARS-CoV-2, severe acute respiratory syndrome coronavirus 2; WHO, World Health Organization; DPP4, dipeptidyl peptidase 4; JNK, c-Jun N-terminal Kinase; JAK, Janus Tyrosine Kinase; STAT, Signal Transducer and Activator of Transcription; HCC, hepatocellular carcinoma; NAFLD, non-alcoholic fatty liver disease; NASH, non-alcoholic steatohepatitis; ALT, alanine aminotransferase; ALP, alkaline phosphatase; AST, aspartate aminotransferase; GGT, gamma-glutamyl transferase.
